# Case Report: Sliding gouty tophi: a case of dynamic ultrasound diagnosis of carpal tunnel syndrome

**DOI:** 10.3389/fsurg.2025.1503085

**Published:** 2025-05-13

**Authors:** Chenchen Qu, Heping Deng, Bo Lu, Yaru Mi, Hui Ye, Yaru Zhu

**Affiliations:** ^1^ Department of Ultrasound Medicine, The Third Hospital of Hebei Medical University, Shijiazhuang, China; ^2^ Trauma Emergency Center, The Third Hospital of Hebei Medical University, Shijiazhuang, China

**Keywords:** gouty tophi, carpal tunnel syndrome, flexor tendon, musculoskeletal ultrasound, median nerve

## Abstract

Carpal tunnel syndrome (CTS) caused by gouty tophi is a rare clinical condition. In this case, ultrasonography clearly identified gouty tophi deposited within the superficial flexor tendon of the right middle finger, resulting in tendon thickening. The thickened tendon was constrained by the transverse carpal ligament, leading to restricted finger movement. Additionally, when the finger was extended, the thickened tendon slid beneath the transverse carpal ligament, triggering severe symptomatic episodes of CTS. This observation provided direct evidence of the underlying etiology of CTS in this patient.

## Introduction

Gout is a common condition caused by the deposition of sodium urate crystals in joints and non-joint structures (1, 2). In gout patients, blood contains a large amount of urate crystals. These crystals circulate and deposit throughout the body, including in joints, ligaments, bursae, tendon sheaths, and subcutaneous tissues, leading to the formation of gouty tophi and inflammatory reactions such as redness, swelling, heat, and pain (2, 3). Carpal tunnel syndrome (CTS), is a compressive neuropathy caused by the compression of the median nerve within the carpal tunnel. It is the most common peripheral nerve entrapment disorder. An increase in the contents of the carpal tunnel or a decrease in its space can increase pressure, leading to compression and injury of the median nerve (4–6).

We report a case of chronic gout in a 49-year-old male patient. The patient had gouty tophi deposits in the superficial flexor tendon of the middle finger, leading to tendon thickening at the wrist. This condition limited movement in the deep surface of the transverse carpal ligament and caused impaired flexion of the middle finger combined with median nerve entrapment. The diagnosis was confirmed by dynamic ultrasonography, and to the best of our knowledge, this specific diagnosis has rarely been previously reported.

## Case report

Seven months ago, a 49-year-old male patient experienced numbness in his right hand without an obvious cause. He received conservative treatment with acupuncture and massage at a local clinic, but there was no significant improvement. Three months ago, his symptoms worsened, including numbness in the radial 3.5 fingers of the right hand and limited flexion and extension of the middle finger. The numbness worsened when the middle finger was straightened and reduced when flexed. In daily life, the metacarpophalangeal joint of the right middle finger is in flexion and it is difficult to actively and passively extend and flex. No obvious muscle atrophy was observed. Phalen's sign was negative, and Tinel's sign was positive. He was admitted to the outpatient clinic with a diagnosis of “peripheral nerve compression syndrome.”

Biochemical examination showed elevated uric acid at 665 μmol/L (normal range: 208–428 μmol/L) and elevated creatinine at 102 μmol/L (normal range: 57–97 μmol/L).

Electromyography showed the distal motor latency (conduction time through the carpal tunnel) of the median nerve was 6.8 ms, the motor conduction velocity of the forearm was 50 m/s, and the amplitude of the CMAP was 4 mv (Patch Electrode). No SNAP waveforms appeared in the median nerve, and neither latency nor wave amplitude could be determined. A small number of positive potentials were seen in the right abductor pollicis brevis, with some MUP time frame widening and wave amplitude increase, but most MUPs were normal. The recruitment pattern showed a mixed phase. The nerve conduction function of the muscle branch above the wrist was not abnormal.

**Figure 1:**
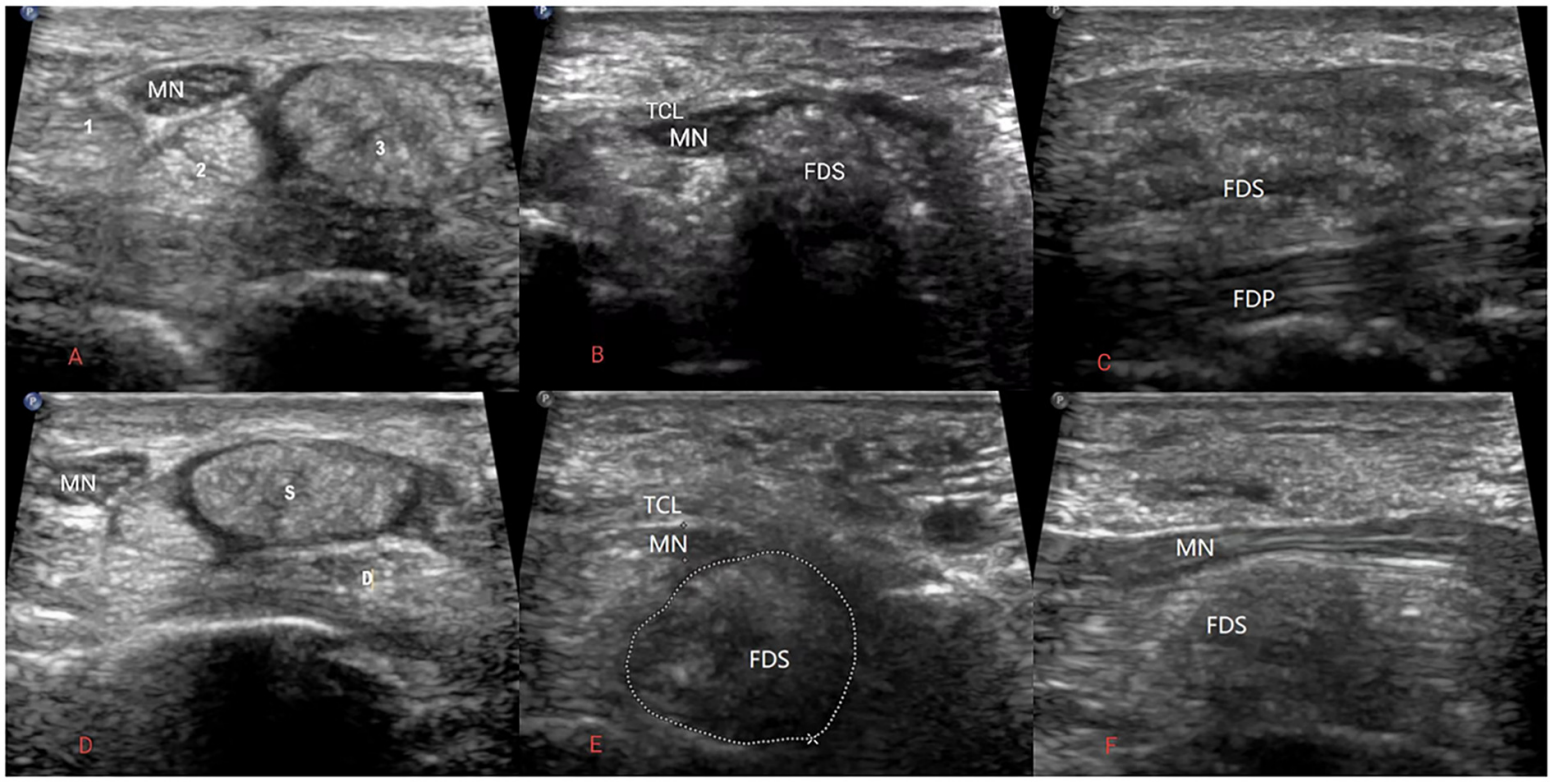
**(A–C)** Flexor tendon and median nerve images of the middle finger in flexed position. **(A)** Transverse section of the proximal carpal tunnel showing the thickest part of the proximal carpal tunnel tendon and swelling of the median nerve. **(B)** Transverse section of the carpal tunnel showing thickening of the tendon and thinning of the median nerve. **(C)** Longitudinal section of the proximal carpal tunnel showing the thickest part of the tendon. **(D–F)** Images of the flexor tendon of the middle finger and the median nerve in the extended position. **(D)** Cross section of the proximal carpal tunnel showing thickening of the tendon and swelling of the median nerve. **(E)** Horizontal transverse section of the carpal tunnel showing the thickest portion of the tendon in the carpal tunnel and significant compression of the median nerve. **(F)** Horizontal longitudinal section of the carpal tunnel showing compression of the thickest part of the tendon and the nerve. (In Figure **(A)** numbers 1.2.3 are the flexor pollicis Longus tendon, flexor digitorum profundus tendon of the index finger and flexor digitorum profundus tendon of the middle finger, respectively. MN, median nerve; TCL, transverse carpal ligament; FDS and S, flexor digitorum superficialis tendon of the middle finger; FDP and D, flexor digitorum profundus tendon of the middle finger).

Ultrasonography of the wrist revealed thickening of the middle finger flexor tendon, with significant thickening of the superficial flexor tendon. The tendon showed disorganized enhancement echoes with a few punctate echoes, and its structure was not clearly defined. Dynamic ultrasound examination revealed that during middle finger flexion (Figures 1A–C), the thickened tendon was restricted by the transverse carpal ligament, primarily located proximal to the ligament. When the middle finger was forcefully extended (Figures 1D–F), the thickened tendon slid distally and positioned itself beneath the deep surface of the transverse carpal ligament. Compressed by the thickened tendon in the carpal canal, the median nerve was flattened and thinned, with a flattening ratio of 4.35. Proximally, the nerve exhibited edema with a cross-sectional area of 18.12 mm^2^. The compression of the median nerve was most pronounced when the middle finger was extended, and the thickened tendon was positioned within the carpal canal. At this position, the thickened flexor tendon of the middle finger and the compressed median nerve were located in the carpal tunnel, causing the patient significant numbness and distension. CDFI showed scattered punctate blood flow signals within the thickened tendon.

**Figure 2:**
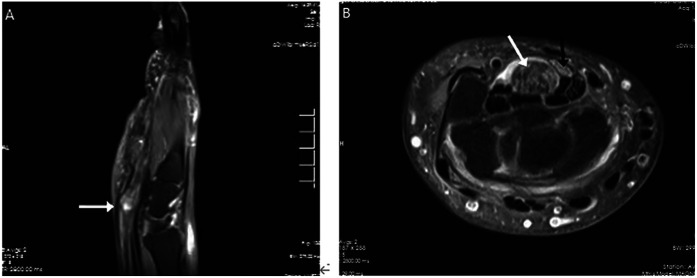
MRI presentation of the patient's right wrist. Part of the superficial flexor tendon of the fingers of the right wrist is significantly thickened (white arrow), with increased T2WI signal and a maximum cross-sectional area of approximately 1.7 cm × 1.3 cm, and the adjacent median nerve is locally compressed with increased signal (black arrow).

MRI revealed significant thickening of part of the superficial flexor tendon in the right wrist, increased T2WI signal, and localized compression of the adjacent median nerve with increased signal (Figure 2).

**Figure 3:**
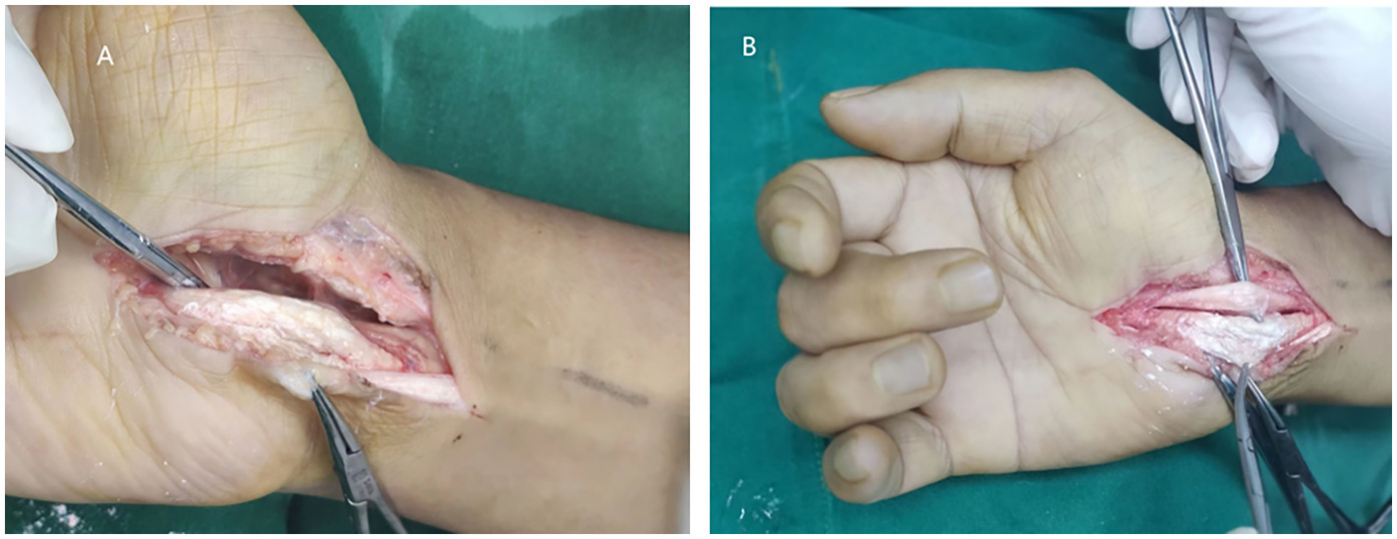
Surgical finding. **(A)** After dissection of the transverse carpal ligament, the superficial flexor tendon of the middle finger was completely exposed, and the tendon sheath was pike shaped. **(B)** Dissection of the tendon sheath of the superficial flexor tendon of the middle finger revealed a large number of milky-white goncholiths within the tendon sheath, and the goncholiths were completely encapsulated by the tendon sheath.

Given the severity of symptoms and imaging findings, surgical treatment was recommended. The patient underwent right carpal tunnel decompression, median nerve release, flexor tendon release, and excision of gouty tophi. Intraoperatively, the median nerve was found to be compressed within the carpal tunnel, while the superficial flexor tendon of the middle finger was irregularly thickened with synovial hypertrophy. A large amount of white, chalky, lime-like deposits was observed between the tendon fibers (Figure 3). To preserve tendon integrity, the thickened portion of the superficial flexor tendon was trimmed and sent for pathological examination, while surrounding adherent tissues were carefully loosened to decompress the median nerve.

**Figure 4:**
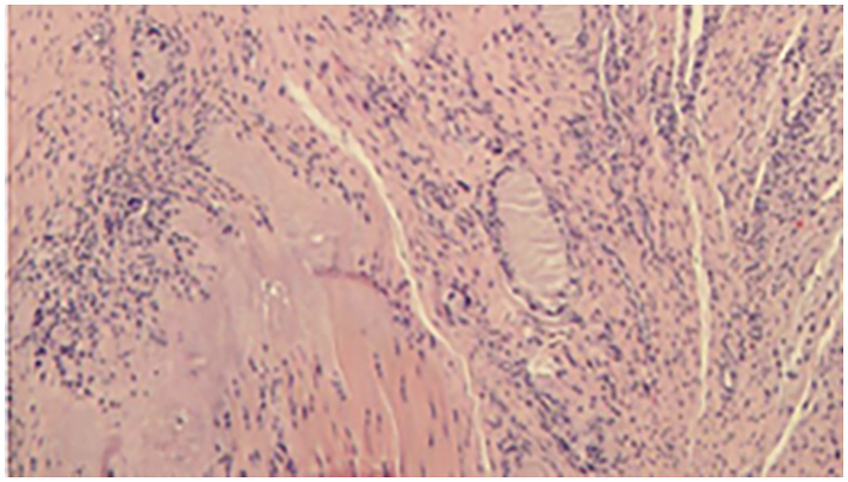
Postoperative pathology suggestive of gout.

Postoperative pathology confirmed gout in the flexor tendon of the right wrist (Figure 4).

Postoperative condition: blood flow to the finger was good, and the patient reported significant relief of numbness. Failure to return to the outpatient clinic for review after surgery as planned. (Due to symptom relief, the patient did not follow-up after surgery.)

## Discussion

This ultrasound demonstrates a case of gouty tophi deposited in the superficial flexor tendon of the middle finger, resulting in limited finger movement and carpal tunnel syndrome (CTS). It is particularly noteworthy that ultrasound can dynamically visualize the sliding of the gouty tophi in the carpal tunnel below the transverse carpal ligament. The ultrasound, combined with the changes in the patient's symptoms at the time, clarified the cause of this patient's CTS and provided an imaging basis for the surgical plan.

CTS caused by gouty tophi is uncommon and predominantly documented as case reports. Hernandez-Cortes et al. (7) reported the first case of CTS with tophaceous infiltration of the flexor tendons as the initial clinical sign of gout, confirmed through surgical findings. Most cases of gout-induced CTS present as a wrist mass and are diagnosed via MRI. Lu et al. (8) described a patient with recurrent CTS and a wrist mass, where MRI indicated that a gouty tophi was compressing the median nerve at the carpal tunnel level. Hao et al. (9) reported tophi deposits in the superficial flexor tendon of a single index finger, with MRI showing gouty tophi compression of the median nerve at the wrist. Occasionally, gouty tophi deposit in the nerve, causing CTS. Zhang (3) and Lee (10) separately reported ultrasonographic findings of high-density deposits in the carpal tunnel of CTS patients, confirmed by surgical findings of gouty tophi beneath the nerve epineurium. In some instances, gouty tophi may be misdiagnosed on MRI, and ultrasound has proven crucial in identifying gout. Rand et al. (11) detailed a case of CTS with a wrist mass initially misdiagnosed as a nerve sheath tumor on MRI, which histologically revealed a gouty origin. Therimadasamy et al. (12) presented a case where CTS combined with a wrist mass was misdiagnosed as a nerve sheath tumor on MRI; surgical exploration confirmed ultrasound findings of gouty tophi in the finger flexor tendons of the wrist. Most peripheral nerve sheath tumors have common ultrasound features, namely hypoechoic, homogeneous, encapsulated, posterior acoustic enhancement and peripheral nerve continuity, and color Doppler ultrasound demonstrates blood flow signals within some lesions (13). Gouty tophi tend to appear on ultrasound as nodules with uneven internal echogenicity with foci of strong echogenicity and posterior acoustic shadowing (14).

This case differs from previously reported gout-induced CTS in several ways. First, there was no palpable mass at the wrist. The patient's gouty tophi deposits were scattered rather than massed at the tendon and were therefore misdiagnosed as idiopathic CTS. Second, this case demonstrates the value of real-time ultrasound assessment in dynamically evaluating tendon motion and its relationship to median nerve compression. The dynamic scan showed that during finger extension, the thickened tendon moved below the transverse carpal ligament, exacerbating the CTS symptoms. This assessment directly demonstrates the underlying pathophysiology and provides a precise anatomical explanation for the development of symptoms.

CTS is more common in females (4, 5), with 50% of cases being idiopathic. Its causes include tissue infiltration, tissue edema, tissue inflammation, and congenital variants (3). Gout is an uncommon couse for CTS. The mechanisms of CTS caused by gouty tophi include thickening of the synovial membrane of the wrist and gouty tophi deposited in the transverse carpal ligament, flexor tendons, tendon sheaths, and even the median nerve (3, 6–12, 15). Besides, studies have shown that gout is more common in males (16, 17). Most gout patients initially present with acute gouty arthritis, primarily affecting the first metatarsophalangeal joint and rarely involving the wrist (1). Through this case, in male patients with a history of gout and relevant laboratory findings, gout should be considered as a potential cause of CTS.

In addition to CTS symptoms, the patient also had impaired extension of the right middle finger, which was overlooked by both the patient and the physician due to the severity of CTS symptoms. High-frequency ultrasound can clearly show tendon morphology during finger flexion and extension, making it a commonly used clinical imaging method. Recurrent episodes of acute gout eventually lead to chronic arthropathy, chronic synovitis, and gouty tophi formation and deposition. When gouty tophi are deposited in flexor tendons, they cause degenerative necrosis of the tendon and eventually tendon rupture, affecting tendon function (7, 15). In this patient, ultrasonography revealed continuous fibers in the superficial flexor tendon of the middle finger of the right wrist, along with scattered and diffusely distributed gouty tophi deposits, resulting in tendon thickening. Real-time dynamic ultrasound examination showed that the thickened tendon was restricted by the transverse carpal ligament, resulting in impaired finger extension. This provided a visual imaging basis for the clinical diagnosis of finger dysfunction.

CTS due to gouty tophi includes both pharmacologic and surgical treatments, both of which have shown good efficacy and are supported by the literature (6, 18). Study has shown that Pegloticase significantly reduces uric acid levels and promotes gouty tophi regression in the treatment of patients with gout (19). In the 6-month randomized controlled trial (RCT), the overall gouty tophi complete remission (CR) rate of 45% in the every 2-week dosing group was significantly higher than in the placebo group (8%). The Open-Label Extension Study (OLE) further demonstrated that with longer treatment, 70% of patients achieved overall gouty tophi CR and 55% of target gouty tophi completely resolved after 1 year. For refractory or gouty tophi-containing gout (e.g., persistently elevated and symptomatic serum uric acid despite optimized treatment with oral uric acid-lowering agents), recombinant uric acid enzyme polyglycolase is now an option (20). However, pharmacological dissolution of gouty tophi can take a long time. Kasper et al. (20) summarized the key indications for surgical treatment of gouty tophi based on the available literature, suggesting that surgical intervention be considered when pharmacological treatment is ineffective and serious complications occur, including poor pain control, nerve entrapment, recurrent infections, sinus tract formation, skin ulceration, functional impairment, joint instability, and limited mobility. In our patient, gouty tophi were deposited in the flexor tendons, resulting in limited extension of the right middle finger and pain and numbness caused by CTS; surgical excision of the gouty tophi and decompression of the median nerve could provide faster and more reliable relief of symptoms.

Intratendinous gouty tophi can present in two patterns: diffusely scattered strong echoes with smaller gouty tophi and extensive deposition, and larger mixed hypoechoic and hyperechoic clusters with more concentrated deposition. The choice of surgical approach should be based on the morphology and localization of the gouty tophi within the tendon. In this case, ultrasound revealed that the gouty tophi were diffusely distributed between the tendon fibers and could not be removed intact. This also explained why the patient did not show a mass on the wrist. Studies have shown that neither trimming nor resection of the superficial flexor tendon of the finger impairs hand movement (6). Therefore, we chose to trim the eroded superficial flexor tendon of the finger while preserving the continuity of the tendon fibers to reduce the tendon volume and relieve median nerve entrapment symptoms. Chen et al. (21) reported the presence of persistent or recurrent carpal tunnel syndrome in 3 out of 20 patients with gouty tophi-induced carpal tunnel syndrome. Postoperative use of uric acid-lowering medications, reduction of alcohol consumption, low purine diet and maintenance of normal serum uric acid levels are needed to prevent disease recurrence.

In conclusion, musculoskeletal ultrasound played a critical role in diagnosing and managing this rare case of gout-induced CTS by identifying intratendinous tophi deposits, providing real-time visualization of tendon movement and nerve compression, aiding preoperative planning for surgical intervention. As dynamic ultrasound techniques continue to evolve, real-time functional imaging is emerging as a valuable tool in diagnosing gout-related CTS. This case underscores the importance of considering gout in the differential diagnosis of CTS, particularly in male patients with hyperuricemia and atypical presentations.

## Data Availability

The original contributions presented in the study are included in the article/Supplementary Material, further inquiries can be directed to the corresponding author.
